# Dating Violence: Idealization of Love and Romantic Myths in Spanish Adolescents

**DOI:** 10.3390/ijerph18105296

**Published:** 2021-05-16

**Authors:** Adelina Martín-Salvador, Karima Saddiki-Mimoun, María Ángeles Pérez-Morente, María Adelaida Álvarez-Serrano, María Gázquez-López, Encarnación Martínez-García, Elisabet Fernández-Gómez

**Affiliations:** 1 Department of Nursing, Faculty of Health Sciences, University of Granada, 52005 Melilla, Spain; ademartin@ugr.es (A.M.-S.); elisabetfdez@ugr.es (E.F.-G.); 2 Hospital Comarcal de Melilla, 52007 Melilla, Spain; karimasaddiki97@gmail.com; 3 Department of Nursing, Faculty of Health Sciences, University of Jaén, 23071 Jaén, Spain; 4 Department of Nursing, Faculty of Health Sciences, University of Granada, 51001 Ceuta, Spain; mgazquez@ugr.es; 5 Department of Nursing, Faculty of Health Sciences, University of Granada, 18016 Granada, Spain; emartinez@ugr.es; 6 Guadix High Resolution Hospital, 18500 Guadix, Granada, Spain

**Keywords:** gender studies, dating violence against women, adolescents, romantic love myths

## Abstract

Dating violence is a significant problem among adolescents. It encompasses a variety of violent behavior, from verbal abuse to physical and sexual abuse, from threats to rape and murder. Among young people, idealization of love and romantic myths are very common as a consequence of our culture and society, which lead them to develop dysfunctional relationships that somehow favor and facilitate partner violence and sexist ideas in daily life. Education is the basic tool to eradicate discrimination and violence against women. The objective of this study is to explore the false myths of romantic love in adolescents and their related factors. A cross-sectional study was conducted with 16–19-year-old teenagers (*n* = 180), through questionnaires and by employing the romantic love myths scale, the ambivalent sexism inventory, and the love attitudes scale. Adolescents accepted to a greater degree the love myths associated with idealization than those related to abuse with scale values of Med = 2.72, SD = 0.55, and Med = 1.34, SD = 0.68, respectively. Designed models predict love idealization on the basis of benevolent sexism (β = 0.03; CI 95% = 0.021–0.039), religion (β = 0.198; CI 95% = 0.047–0.349), passionate love (β = 0.038; CI 95% = 0.015–0.061), practical love (β = 0.024; CI 95% = 0.001–0.047), and friendly love (β = 0.036; CI 95% = 0.014–0.058). Hostile sexism and undergraduate studies were associated with the myths that relate love and abuse (β = 0.19; CI 95% = 0.007–0.031, β = 0.208; CI 95% = 0.001–0.414, respectively).

## 1. Introduction

Intimate partner violence (IPV) is one of the most significant representations of inequality, subordination, and power relationships by men against women [1]. The percentage of women around the world affected by it reaches 30%, thus constituting an important public health issue occurring in every country, culture, and social class, with serious consequences on women’s health, both physical and mental [2].

In Spain, according to the data published by the Government Delegation for Gender Violence in 2020, 45 women were murdered by their partners or former partners and 113,615 intimate partner violence cases were reported [3].

Studies have revealed that, despite the efforts made, violence against women still exists among Spanish teenagers and schoolchildren [4,5,6]. From 2013, the year in which cases of teen dating violence started being recorded in our country, 37 murders of young women by their partner or former partner have been registered [7]. Diaz-Aguado et al., in their study “La situación de la violencia contra las mujeres en la adolescencia en España” (Violence against women in adolescence in Spain) point out that the risk of all kinds of violence, both verbal abuse and threats, physical and sexual, as well as rape and murder, increases during dating. Among 16–24-year-old girls who state having had a romantic relationship, 2.5% of them suffered physical violence compared to the 0.8% reported by 25-year-old or older women. Likewise, the estimated prevalence in both age groups of sexual violence is of 3.7% and 1.1%, respectively; of psychological violence involving control, of 17.3% and 5.9%; and of psychological and emotional abuse, of 11.6% and 5.0%. The latter is especially frequent among adolescents, being reported by 20% of 16–17-year-old women who have been in a relationship, diminishing as age increases [5]. Relevant differences in psychological consequences were also observed, where 80.8% of young women have experienced them, compared to 68.3% of older women. In addition to anxiety or anguish, wanting to cry for no reason, irritability, and mood swings, they report a higher level of suicidal ideation [5]. Within the academic context, these women show more difficulty in their studies, their integration with peers, and self-esteem, compared to those who have not suffered IPV [8]. However, they resort to police or courts in a lesser degree to report the violence suffered (14.5% vs. 22.6%), or to request help services (health, psychological, social, legal assessment services, etc.) to face the consequences thereof (27.3% vs. 33.3%) [5].

Adolescence is a period in which several changes occur at biological, psychological, and social levels. Sexual attraction and relationships start taking place, and the idealization of love and the existence of romantic myths are very common [9]. Romantic love is derived from the myth of the Androgyne described in Plato’s The Banquet [10], which justifies the bases of the naturalness and universality of love. Nevertheless, today it is based more on polite love and the need to live with someone who completes our existence [11]. Romantic love myths mainly lie in the obsessive search for the perfect partner who will make us totally happy, so that society has come to consider that suffering is natural if such complement is not found [11].

For the patriarchal society, being a woman, and its related female roles, is associated with being a wife and a mother and, therefore, with the task of providing care, affection, and love to others [11]. Moreover, from several religious sectors of the patriarchal society, the passive role and the role of subordination of women towards men have been encouraged and reinforced. The media also promote the idea that romantic love is the ideal for unique and true love [12]. Thus, in order to attain such love, young women may consider that they should surrender to their partners totally, just like a mother love [13]. In this way, romantic myths at such ages are deemed representations of so-called true love [14]. But the existence of these myths in a relationship may easily lead to episodes of gender violence which, however, may be understood as acts of love [12,13,15].

On the other hand, it has been widely documented that sexist attitudes help perpetuate gender inequality [16]. Glick and Fiske state that sexism is based on three ideas: dominating paternalism (women are weaker and inferior to men); competitive gender differentiation (women are different and do not have the necessary characteristics to govern social institutions, family and home being their places); and heterosexual hostility (women, due to their sexual power, are dangerous and have the capacity to manipulate men) [17]. These authors also differentiate two components which, although related, are clearly distinguished: hostile sexism and benevolent sexism. Hostile sexism, or traditional sexism, considers women inferior and weak with respect to men. Benevolent sexism is defined as a set of attitudes towards women based on a stereotyped vision limited to certain roles, but which has a positive emotional aspect for the receiver [17,18].

Education is essential to prevent partner violence, since it is the basis of knowledge and it allows for the development of skills which train teenagers to make their own decisions and undertake their own responsibilities. Such early ages are favorable for modifying rigid teachings, myths, taboos, beliefs, and conduct which may have had an influence on their education [19,20,21,22]. That is why, in the last several years, multiple educational interventions have been developed with the aim of promoting gender equality; preventing aggressive behavior among peers, both within and without social media; as well as addressing sexist attitudes and romantic myths among adolescents [23,24]. Such an example is the program “Developing healthy and egalitarian adolescent relationships” (DARSI) implemented in the Valencian Community (Spain), which showed an association between this type of intervention and a significant decrease in aggressive conduct. The efficacy of the program according to Wilks’ Lambda distribution was 0.656, F (12, 338) = 6.608, *p* < 0.001, with a large effect size, η^2^ = 0.190) [25].

However, apart from educational institutes’ involvement in and commitment to partner violence prevention, their interaction with court, social, and health systems is also necessary [26,27,28]. The role of the school nurse is strategic in education centers, both to detect cases of dating violence, provide timely health attention, and design strategies that shall help prevent and eradicate such phenomena [29]. Nevertheless, the information related to the persistence of romantic myths in adolescents and their related factors is still incipient. The objective of this study is to explore the false romantic myths in young people aged 16–19 and their related factors.

## 2. Materials and Methods

### 2.1. Data Collection

This is a cross-sectional study by means of an anonymous questionnaire, self-completed online by 16–19-year-old adolescents in April 2020 in a city in Southern Spain. The number of students studying in the last year of secondary school and the first year of study at a university in the city were 299 and 91, respectively. By means of non-probability quota sampling by course, with a confidence level of 95% and maximum estimation error of 7%, a sample size of 180 adolescents was estimated. Said method was chosen to guarantee the proportionality of individuals with undergraduate and graduate studies regarding distribution of students enrolled in the centers selected for the study. All enrolled students had the same opportunity to access the questionnaire.

### 2.2. Measurement Tool

For collection of data, Bosch et al.’s (2008) romantic love myths scale [30], Glick and Fiske’s (1996) ambivalent sexism inventory [17] and Hendrick, Hendrick, and Dick’s (1998) love attitudes scale [31] were employed. In addition, variables such as gender, age (years old), religion (being a believer or a non-believer), romantic status (in a relationship and not in a relationship), and level of study (undergraduate and graduate) were collected.

The romantic love myths scale [30] was validated in Spanish by Bosch et al. It comprises 10 items grouped by two dimensions: love idealization (8 items) and love–abuse relationship (2 items). The answer format is Likert type, 1 = totally disagree and 5 = totally agree. The total scale showed good reliability (Cronbach’s alpha = 0.64), being 0.69 for the love idealization subscale, and of 0.77 for the love–abuse link subscale [32]. Lemus et al. [33] adapted to Spanish the ambivalent sexism inventory [17], composed of 22 items with two dimensions, hostile sexism and benevolent sexism, of 11 items each. The Likert type answer format ranges from 1 = totally disagree to 5 = totally agree. Measurements of the general scale as a measure of ambivalent sexism show a Cronbach’s alpha of 0.81, which in the hostile sexism subscale is 0.84, and in benevolent sexism, 0.77 [33]. Finally, the love attitudes scale designed by Hendrick, Hendrick, and Dick and validated in Spanish by Rodríguez-Castro et al., 2013 [34] is composed of 18 items grouped by six dimensions, corresponding to six types of love: romantic love, playful love, friendly love, practical love, obsessive love, and altruistic love [31]. Likewise, it uses Likert type answers where 1 = totally disagree and 5 = totally agree, the scale reliability ranging from 0.88 for altruistic love to 0.68 for practical love [34].

### 2.3. Data Analysis

The sample was described by means of frequencies and measures of central tendency (mean and standard deviation) pursuant to the quantitative or qualitative nature of the variables analyzed. Later, bivariate analyses were conducted for love idealization and for the love–abuse link, the two components of romantic love myths, considering as independent variables sociodemographic factors (age, gender, level of study, romantic status, and religious beliefs), the dimensions of the ambivalent sexism scale (benevolent sexism and hostile sexism) and love attitudes (passionate love, playful love, friendly love, practical love, obsessive love, and altruistic love). For contrasting means, the Student’s t parametric test was applied and the effect size was calculated by means of Cohen’s d. Furthermore, Pearson’s correlation analysis was carried out on both dimensions of romantic love myths scale with age, dimensions of love attitudes and ambivalent sexism scales, establishing the level of statistical significance at a value of *p* < 0.05.

Finally, two models of stepwise multiple linear regression were designed by introducing all the independent variables, estimating the beta values, standard error, and their confidence intervals at 95%. The linearity and independence of residuals were verified by means of the Durbin–Watson statistic, thus determining acceptable values of independence of less than 2.5.

Every analysis has been conducted using the Statistical Package for the Social Sciences (SPSS) program, version 25, (IBM, New York, NY, USA, for Mac).

### 2.4. Ethical Considerations

The study complies with good clinical practice regulations, set forth in European Directive 2001/20/EC, and in Law 14/2007, of 3 July, on biomedical research. Treatment of personal data in health research is governed by the provisions of Organic Law 3/2018 of 5 December, on the protection of personal data and guarantee of digital rights. Participants were informed of the objectives of the study and provided their consent to participate by checking a specific box.

## 3. Results

The sample included 184 students with a mean age of 17.6, SD = 1.15 years and a female ratio of 74.5%. As regards level of study, 121 (65.8%) were undergraduate students, 62% (*n* = 114) were not in a romantic relationship, and 128 (69.6%) declared themselves believers (Table 1).

Table 2 shows the mean of scores obtained in the different dimensions of the romantic love myths, ambivalent sexism, and love attitudes scales. Love idealization and benevolent sexism reached the highest scores of 2.72 (0.55) and 2.25 (0.65), respectively. Playful love is the one which, less frequently, participating adolescents state they feel, with a score of 2.11 (0.96).

Table 3 shows the results of bivariate analyses for the two dimensions of the romantic love myths scale. Among those who declared themselves believers, a score of 2.83 is observed in love idealization with statistically significant differences and a mean effect size with respect to non-believing adolescents (*p* = 0.001; Cohen’s d = 0.683). Likewise, benevolent sexism was positively and moderately correlated to this dimension (r = 0.487; *p* = 0.01), while practical love, passionate love, hostile sexism, friendly love, obsessive love, and altruistic love did so weakly (*p* = 0.01). Regarding the love–abuse link dimension, men and adolescents studying undergraduate studies were the ones who showed higher scores although with a weak effect magnitude (*p* = 0.033; Cohen’s d = 0.361 and *p* =006; Cohen’s d = 0.105, respectively). Hostile sexism was slightly associated with this component (r = 0.260; *p* = 0.01), followed by altruistic love, playful love, and obsessive love (*p* < 0.05). Although weakly, it is observed that the older the individual, the less the association with love–abuse (r = −0.166; *p* = 0.05).

Table 4 shows the final regression model for the love idealization dimension with the five predictor variables that explained the 39.5% variability. In this sense, adolescents who are believers, with a tendency toward benevolent sexism and with a vision of passionate, practical, and friendly love are the ones who tend to idealize love.

For the love–abuse link dimension, the predictive model was composed of the hostile sexism and undergraduate studies variables, which explained the 8.7% variability (Table 5). It was undergraduate students with a tendency toward hostile sexism who showed a tendency toward the love–abuse link.

## 4. Discussion

This study contributes significant data on the influence of sexism on adolescents, as well as on their beliefs and attitudes towards love in different academic contexts, thus increasing the empirical evidence of such knowledge [12,35,36].

Our results show that love myths related to the idealization of love are more accepted by adolescents than those related to abuse. Moreover, more sexist benevolent attitudes are observed than hostile attitudes, as previously documented in populations with similar sociodemographic characteristics [31,35,36]. They also reveal the relationship between both concepts; that is, an increase in sexist attitudes, whether hostile or benevolent, seems to lead to the mythification of love. During adolescence, Carbonell and Mestre identified these attitudes as risk factors for the establishment of unbalanced relationships and with a tendency toward abuse [37].

Among all love attitudes analyzed, the passionate vision was highlighted above all others, followed by the friendly one, as regards the establishment of a long-term commitment, and of the practical one or search for the perfect love, being the product of the combination of requirements more than of own feelings. Such results coincide with those reported by Moyeda et al., who interpret them from the perspective of social models imposed through different media and institutions [38].

With respect to gender, boys and girls in this study had a similar romantic perception, as found by Rodríguez and Alonso [39], although such findings disagree with those of other authors. In this sense, it is reported that women may tend to surrender totally to their partners, while men have a more demanding attitude [13,37]. Cava et al. [40] also demonstrated differences between boys and girls. Although romantic love myths were a significant predictor of cybernetic control victimization (ways of abusive control of victims to monitor their social relationships and what they are doing at any time) both in boys and girls, said authors hold that believing such myths could have had a higher impact on girls. On the other hand, in both genders, romantic myths were a significant positive predictor of cybercontrol and cyber-aggression victimization (behavior involving harm to victims through direct attacks, for instance, threats, insults, or disclosure of private information). Nevertheless, its weight has better predicted cybercontrol victimization in adolescent girls. We do observe higher scores in the love–abuse link among adolescent boys, which result coincides with other publications [33,41]. Such attitudes denote the internalization of violence within the romantic relationship context from an early age, which is really disturbing, especially in a society where formal or legal equality is a reality, but, however, to reach actual gender equality, it seems evident that more efforts are still required [42].

Age was inversely related to the love–abuse link, so that as it increases, the association diminishes. Although the age margin analyzed is narrow, this finding may be indicative of a tendency toward the establishment of healthier romantic relationships as adolescents mature [37,43,44].

Upon consideration of the level of study, undergraduate students supported slightly more negative attitudes in the love–abuse link than graduate students. Wang [45] observed that as the educational level of participants increased, attitudes and perceptions towards partner violence had no significant effect. In contrast, Navarro-Pérez et al. [44] point out that formal education has a direct influence on sexist attitudes; in the intervention performed, sexism was reduced between 6 and 12%.

The sociodemographic variable with higher relevance in our study was, undoubtedly, considering yourself a believer. This aspect has an impact on both dimensions, in particular, on love idealization with a mean effect size. People with religious beliefs may also show more traditional lifestyles tending toward a higher normalization of such myths. In Prina and Schatz-Stevens’ study [46], religiousness was also a strong predictor of sexism both in the U.S. and the Italian populations (β  = 0.25, *p* < 0.001; β  = 0.31, *p* < 0.001, respectively) as well as of the acceptance of the violence myth (β = 0.21, *p* < 0.05; β  = 0.13, *p* < 0.01). Other studies explored the link between religious affiliation and violence against women [47,48,49]. Ghanim [49] compared the relationship between Christians and Muslims, and different types of violence against women, according to opinions of women themselves. Muslims experienced higher prevalence in all types of violence (physical and/or sexual, and psychological) compared to Christian women, which may be taken into account to locate populations with a higher risk of suffering this kind of violence.

Our predictive model, obtained for love idealization, was determined by benevolent sexist conducts, being a believer, and by attitudes favoring passionate, practical, and friendly love, which findings coincide with those documented by other authors. Gul et al. [50], in their review of literature, concluded that women find men with benevolent sexism attitudes attractive because they felt they were willing to protect, provide, and commit, despite being aware of the harmful consequences. Lelaurain et al. [51] reported that participants more inclined to romantic love were prone to blaming victimized women and to exonerating the batterer, which means a link among romantic love, ambivalent sexism, and domestic violence myths. Furthermore, it has been widely documented that love styles are learnt through cultural rules and practices in the relationship. In traditional cultures, the practical love style seems to be less valued by considering that this feeling arises more from an emotional act than from a logical one [31,52,53]. Therefore, it is possible to think that more religious societies will tend to perpetuate myths related to the idealization and search for perfect love like passionate, friendly and practical love.

Hostile sexism and a lower level of studies, in our predictive model, were slightly associated with the love–abuse link. Hostile and benevolent sexism are complementary ideologies that prevail in cultures, and both predict gender inequalities [19]. In Spain, it has been observed that within a short time period, sexist attitudes have been increasing significantly among young people. Since the purpose of education in equality is currently a priority in our educational context, it may not, however, be producing certain results such as reducing hostile sexism, at least among participants of this study. Although we observe a slight association between a lower level of study and love–abuse, we coincide with Bosch et al. [54] when pointing out that associating a low sociocultural level with partner violence is a misogynistic myth traditionally used to justify such violent conducts. In general, this study is in line with those concluding that the perpetuation of romantic love myths helps maintain gender stereotypes and inequality between men and women, thus justifying the violence suffered by women in their romantic relationships [55,56].

The relationship of care between nurses and patients makes it possible to value a person comprehensively, their family and social environment, and the consequences for their children. In this sense, the role of a nurse includes care and training and education on an equal footing regarding adults and, especially, children and young people. Since they provide health education in sexual intercourse and consent, nurses may help promote healthy relationships among adolescents. This guarantees that young people know any resources and support available in order to avoid sexual abuse and violence. Furthermore, nurses facilitate access to services for victims of abuse [57]. The figure of the school nurse could, by means of workshops, design strategies that shall help prevent and eradicate such phenomenon [29].

### Study Limitations

This study has several limitations. First of all, the type of quota sampling may have inclined participation towards participants more aware of gender equality, which would include a classification bias. Moreover, the low participation of boys, regardless of reflecting their low interest in this topic, may have minimized the associations found. However, in general, the results observed are in line with other studies, which contributes theoretical consistency to the study. To overcome such limitations, in subsequent research, a higher effort should be made to achieve gender-balanced participation.

Although the representation of students has been satisfactory and, therefore, the internal validity of the study may be deemed adequate, in order to generalize our results, the number of educational centers should be increased to increase external validity and, therefore, its generalization to other contexts.

Lastly, the existence of social desirability bias in answers should not be eliminated, since we addressed especially sensitive topics like love myths and sexism. To control it, the anonymity of the students, who volunteered for the study, was guaranteed.

## 5. Conclusions

We have observed that adolescent students tend to accept love myths related to idealization of love more than those related to abuse. Such idealization of love was related to practicing a religion, and not to gender, level of study, or romantic status. Undergraduate boys showed attitudes more related to love–abuse.

Benevolent sexism, religion, passionate love, practical love, and friendly love predicted a higher idealization of love, while hostile sexism and undergraduate studies acted as factors predicting love–abuse.

Awareness of these factors could be useful in designing socio-educational strategies addressing love myths and sexist attitudes still persistent among adolescents in our region. School nurses have an important role to play in education centers as professionals directly involved in the attention and prevention of violence against women in the relationship context, mainly at an early age.

## Figures and Tables

**Table 1:** Participants’ sociodemographic characteristics.

**Variables**	**Participants**
	(*n* = 184)
	M (SD)
**Age (years old)**	17.6 (1.15)
	***n*** **(%)**
**Gender**	
Male	47 (25.5)
Female	137 (74.5)
**Level of study**	
Undergraduate	121 (65.8)
Graduate	63 (34.2)
**Romantic status**	
Not in a relationship	114 (62)
In a relationship	70 (38)
**Religion**	
Believer	128 (69.6)
Non-believer	56 (30.4)

M = mean; SD = standard deviation; *n* = frequency; % = percentage.

**Table 2:** Descriptive values of romantic love myths, ambivalent sexism, and love attitudes scales.

	M (SD)
**Romantic love myths**	
Love idealization	2.72 (0.55)
Love–abuse link	1.34 (0.68)
**Ambivalent sexism**	
Benevolent sexism	2.25 (0.65)
Hostile sexism	1.99 (0.72)
**Love attitudes**	
Passionate love	3.3 (0.96)
Playful love	2.11 (0.97)
Friendly love	3.16 (0.01)
Practical love	3.16 (0.1)
Obsessive love	2.41 (0.85)
Altruistic love	2.59 (0.97)

M = Mean; SD = Standard Deviation.

**Table 3:** Bivariate analysis for dimensions of the romantic love myths scale.

	Love Idealization			Love–Abuse Link		
	M (SD)	*p*	Cohen’s d	M (SD)	*p*	Cohen’s d
**Gender**						
Male (*n* = 47)	2.72 (0.56)	0.960		1.52 (0.77)	0.033 *	0.361
Female (*n* = 137)	2.73 (0.54)			1.28 (0.64)		
**Level of study**						
Undergraduate (*n* = 121)	2.74 (0.53)	0.501		1.44 (0.74)	0.006 **	0.105
Graduate (*n* = 63)	2.69 (0.57)			1.15 (0.50)		
**Romantic status**						
Not in a relationship (*n* = 114)	2.71 (0.53)	0.743		1.39 (0.75)	0.239	
In a relationship (*n* = 70)	2.74 (0.58)			1.26 (0.55)		
**Religion**						
Believer (*n* = 128)	2.83 (0.53)	0.001 **	0.683	1.38 (0.75)	0.288	
Non-believer (*n* = 56)	2.48 (0.51)			1.26 (0.48)		
	**r**	***p***		**r**	***p***	
**Age**	0.040	0.588		−0.166	0.025 *	
**Benevolent sexism**	0.487	0.001 **		0.077	0.297	
**Hostile sexism**	0.268	0.001 **		0.260	0.001 **	
**Passionate love**	0.301	0.001 **		−0.062	0.4	
**Playful love**	0.070	0.344		0.177	0.017 *	
**Friendly love**	0.254	0.001 **		0.001	0.992	
**Practical love**	0.331	0.001 **		−0.075	0.313	
**Obsessive love**	0.252	0.001 **		0.157	0.033 *	
**Altruistic love**	0.216	0.006 **		0.187	0.011 *	

M = Mean; SD = Standard Deviation; r = Pearson Correlation Coefficient; * *p* < 0.05. ** *p* < 0.01. Cohen’s d = Effect size.

**Table 4:** Multiple linear regression model for love idealization.

Variable	β	Error Dev.	95% CI	
Benevolent sexism	0.03	0.005	0.021	0.039
Believer	0.198	0.076	0.047	0.349
Passionate love	0.038	0.012	0.015	0.061
Practical love	0.024	0.012	0.001	0.047
Friendly love	0.036	0.011	0.014	0.058

Durbin Watson Test = 1.709; F = 23.24; *p* < 0.001.

**Table 5:** Multiple linear regression model for the love-abuse link.

Variable	β	Error Dev.	95% CI	
**Hostile sexism**	0.19	0.006	0.007	0.031
**Undergraduate**	0.208	0.105	0.001	0.414

Durbin Watson Test = 1.882; F = 8.68; *p* < 0.001.
